# Essential Thrombocythemia in Children and Adolescents

**DOI:** 10.3390/cancers13236147

**Published:** 2021-12-06

**Authors:** Maria Caterina Putti, Irene Bertozzi, Maria Luigia Randi

**Affiliations:** 1 Clinic of Pediatric Hematology-Oncology, Department of Women’s and Children’s Health, University of Padua, 35128 Padua, Italy; mariacaterina.putti@unipd.it; 2 First Medical Clinic, Department of Medicine—DIMED, University of Padua, 35128 Padua, Italy; irene.bertozzi@unipd.it

**Keywords:** pediatric myeloproliferative neoplasms, thrombocytosis, ET diagnosis, ET treatment

## Abstract

**Simple Summary:**

Among chronic Ph-negative myeloproliferative neoplasms, essential thrombocythemia is found in children with low but increasing incidence. The diagnostic and clinical features do not completely overlap with ET of adult age. A significant number of cases, in fact, do not meet the criteria of clonality, and many cases require extensive clinical evaluation to exclude secondary, reactive forms. Therefore, histological analysis of bone marrow biopsy is necessary, and its use should be enforced. The clinical course appears to be more benign, at least within the first decades of observation, with the incidence of thrombotic events being much lower than in adults (4 % vs. 30%). Hemorrhages are mostly irrelevant. Therefore, the management should be carefully adapted to the individual patient, balancing the risk of future complications with long-term collateral effects of any drug. This review analyzes the peculiarities of the disease facing similarities and differences with adult scenarios.

**Abstract:**

This paper reviews the features of pediatric essential thrombocythemia (ET). ET is a rare disease in children, challenging pediatric and adult hematologists alike. The current WHO classification acknowledges classical Philadelphia-negative MPNs and defines diagnostic criteria, mainly encompassing adult cases. The presence of one of three driver mutations (*JAK2*V617F, *CALR*, and *MPL* mutations) represent the proof of clonality typical of ET. Pediatric ET cases are thus usually confronted by adult approaches. These can fit only some patients, because only 25–40% of cases present one of the driver mutations. The diagnosis of hereditary, familial thrombocytosis and the exclusion of reactive/secondary thrombocytosis must be part of the diagnostic process in children and can clarify most of the negative cases. Still, many children present a clinical, histological picture of ET, with a molecular triple wild-type status. Moreover, prognosis seems more benign, at least within the first few decades of follow-up. Thrombotic events are rare, and only minor hemorrhages are ordinarily observed. As per the management, the need to control symptoms must be balanced with the collateral effects of lifelong drug therapy. We conclude that these differences concert a compelling case for a very careful therapeutic approach and advocate for the importance of further cooperative studies.

## 1. Introduction

Essential thrombocythemia (ET), one of the Ph-negative Chronic Myeloproliferative neoplasms (MPNs), develops as an acquired clonal defect of myeloid precursor cells driving uncontrolled myeloid proliferation [1]. MPNs are characterized by elevated blood cell counts, with every lineage being distinctive to the various entities, albeit with some degree of overlapping. Within MPN, ET is characterized by high platelet counts, a finding which can also be found in polycythemia vera (PV), myelofibrosis (MF) and chronic myeloid leukemia (CML) [2]. Diagnosis of ET as well as of other MPNs has long been addressed by adult hematology and was recently confirmed within the WHO classification of myeloid neoplasm [3,4], gaining from the insight given by the identification of driver mutations and peculiar histological patterns (Table 1).

ET is a rare disease in middle–advanced age and is even rarer in children [5]. Diagnosis and treatment of this clonal condition in pediatrics face both certainties and many dilemmas. First, the definition of thrombocytosis has been the matter of debate in children, because differences in platelets counts are seen in various ages and only a few studies have analyzed the issue. In fact, 30% of neonates and infants show high values, especially in the case of low-weight babies, [6,7]; an upper limit of 600 × 10^9^ is considered normal in these children [8,9]. In older children and adolescents, the upper normal limit (95 percentile) of 450,000 has been identified [10], as suggested by the Italian Association for Pediatric Haematology Oncology (AIEOP) guidelines [11] and by other classifications of thrombocytosis [12]. Nonetheless, transient conditions and secondary reactive thrombocytosis are very common findings in pediatrics and require a thorough approach even when clear causes cannot be identified. The classical partition between primary and secondary/reactive thrombocytosis does not apply straightforwardly to children. Moreover, a third category of hereditary or familial thrombocytosis (HT) is unique to young age and must be ruled out [13]. Only in recent years has interest been focused on pediatric primary thrombocytosis and, in particular, on ET. Some papers [14,15,16,17,18] suggest that ET in children is different than in adults, with lower incidences of thrombotic and hemorrhagic complications and scarce or no evolution into myelofibrosis and acute leukemia. This poses questions regarding the opportunity of adapting the available hematological and management criteria to pediatric patients. The present paper reviews current ideas on different manifestations of thrombocytosis in children and the main differences between primary and secondary thrombocytosis. Diagnostic and therapeutic implications are discussed.

## 2. Hereditary/Familial Thrombocytosis (HT)

Hereditary and familial thrombocytosis are extremely rare. They share the same clinical aspects of primary thrombocytosis, comprising splenomegaly and the risk of thrombotic complications. Most of them have a Mendelian inheritance, are polyclonal and only affect the platelet lineage [13,19,20]. Affected genes are usually *TPO* or more frequently, its receptor c-MPL; gain of function mutations in the *TPO* gene have been described [21] and result in the proliferation of megakaryocytes, the activation of platelets and the significant overexpression of membrane molecules [22]. A founder effect found in families from Central Italy might explain the different percentages of non-clonal cases in Giona’s series [23].

## 3. Reactive/Secondary Thrombocytosis (ST)

Many children have transitorily increased platelet counts, mostly related to inflammatory conditions (Table 2). Therefore, an isolated finding of thrombocytosis is usually not clinically relevant in children. In contrast, if thrombocytosis is confirmed over at least 3–6 months, it may have a different pathogenesis and clinical impact [12,15].

Megakaryopoiesis is controlled by various growth factors and cytokines. By far, the most essential growth factor is thrombopoietin (TPO), which is produced constitutively by the liver [24], but also by marrow stromal cells and by the kidney. TPO intervenes both in stem cell differentiation and in all stages of megakaryocytes maturation [25], through the binding to its receptor c-MPL and the activation of kinases of proliferative pathways (JAK, STAT1, STAT2, PI3K and MAPK). Its production is regulated by the bulk of circulating platelets and of marrow megakaryocytes.

Hepatic TPO mRNA expression is increased with inflammation [26]. Moreover, many interleukins (IL) involved in the inflammation processes, mostly IL-6 and IL-11, can stimulate the platelet production in the liver [27,28]. These processes are highly active during childhood: ST is reported in 6–15% of hospitalized children, and up to 1% of children in intensive care units show platelet counts exceeding 1000 × 10^9^/L [5,29,30,31,32,33,34,35].

Infections, both bacterial and viral, are common causes of thrombocytosis in infants (75% of cases [31]), and children (47% [33]), mostly of the respiratory and urinary tracts. All other pediatric inflammatory conditions are associated with ST [34], in particular those seen in young children, such as Kawasaki disease [36], Schoenlein–Henoch syndrome, coeliac disease as well as rheumatic diseases and damages to tissues due to trauma, surgery or burns [8]. Many neoplasms (including acute leukemias) are also described to cause ST as well as chemotherapeutic drugs, steroids or adrenaline. Iron deficiency is a known cause of ST (5–12% [37]), although its precise mechanisms remain to be elucidated. Studies on megakaryocytes demonstrated that iron is essential for their proliferation as for other cells [38], and iron replacement resolves thrombocytosis [39].

The clinical relevance of ST is still debated. Platelet count can be regarded as an “acute phase” reactant [40], and has been shown to correlate with prognosis, mainly the prolongation of hospital staying. Most authors agree that ST is a collateral effect of the underlying disease and does not represent a risk for the patient. In fact, neither thrombotic nor hemorrhagic complications were seen in more than 4000 reported children with ST [32]. In 855 children affected by extreme thrombocytosis, Thom et al. reported 22 thromboses, all related to underlying conditions, and no hemorrhages [33].

Finally, asplenic patients commonly display an increased platelet count. Splenic atrophy or agenesia are extremely rare conditions, while splenectomies are performed in a wide spectrum of hematologic disorders: hereditary and autoimmune hemolytic diseases, thrombocytopenia, myeloproliferative diseases, leukemias and lymphomas [41], and a splenectomy could also be necessary after trauma [42]. Thrombocytosis tends to reduce within 6–12 months after splenectomy, sometimes never resolving. It has been shown that the thrombotic risk is likewise related to the underlying condition; in particular, it is higher in the case of lymphomas and myeloproliferative disorders, as well as in hereditary spherocytosis (HS) [41]. The use of antiaggregant therapy or other prophylactic measures are therefore to be used according to the primitive diagnosis, not only to the platelet count. In conclusion, ST does not usually require treatment, and the platelet count alteration usually normalizes with the resolution of the causal disease. Children with persistent thrombocytosis, but without any recognizable cause, should be investigated for the possible diagnosis of primary clonal or familial condition. 

## 4. Essential Thrombocythemia (ET)

In ET (also commonly assumed as primary thrombocytosis), the increase in platelet count depends on a clonal disease of the bone marrow. These forms were grouped as myeloproliferative diseases and were later considered myeloproliferative neoplasms by the 2016 WHO classification [3,4] and comprise different entities, such as BCR-ABL-positive chronic myeloid leukemia, chronic eosinophilic leukemia, mastocytosis, some myelodysplastic syndromes, and the three entities within BCR-ABL-negative chronic MPN, i.e., ET, PV and MF. Thrombocytosis is the main hematological feature in ET, which, albeit typical of advanced age, is the most common MPN of childhood [5,6,7,8]. ET has an estimated incidence of 1 per 10 million annually [5]. However, ET is now recognized more frequently than in the past and an incidence of 0.6/100,000/year has been reported in children and young people under the age of 20 [43], probably due to the increased number of automated blood counts performed in children [18], and case series, not only case reports, have been published in recent years [8,15,16,17,18,43,44].

## 5. Clinical and Molecular Biology of Adult ET

Our understanding of the pathophysiology of MPNs has improved dramatically since the recurrent molecular abnormalities in these disorders became known. In 2005, the era of the molecular diagnosis of MPN began with the discovery of Janus kinase 2 mutation (*JAK2*V617F) [45,46,47,48,49], present in most PV, more than half of ET and MF. Myeloproliferative leukemia virus oncogene (*MPL*) mutations, mostly at position 515 (the most frequent one is *W515L*), were identified in ET in 2006 [50,51]. Finally, in 2013, mutations (insertions and deletions) of the calreticulin gene (*CALR*), [52,53] were found in ET and in some MF.

These are all acquired, somatic mutations of stem cell progenitors, driving the activation of the same JAK2 pathway and the uncontrolled proliferation of different myeloid lineage cells. They are usually mutually exclusive [54,55]. *JAK2*, *MPL* and *CALR* mutations are common in adult ET, with their frequency being around 50–60%, 1–5% and 15–25%, respectively. About 5–10% of adult ET patients do not present any of these molecular markers and are referred to as triple-negative (3NEG) ET [56,57].

Clinically, mutated *CALR* cases have been associated with younger age, male sex, higher platelet count, lower hemoglobin level and lower leukocyte count than *JAK2V617F* cases [58]. The presence of driver mutations does not affect survival in adults [55]. The thrombotic risk is higher in *JAK2V617F* but not in *CALR*-mutated ET [59,60]. *MPL* mutations are associated with clinical features similar to *CALR*, but without pre-fibrotic histological changes [61]. In selected cohorts of true-ET, the 3NEG mutational status seems to correlate with a remarkably favorable overall survival rate and few cardiovascular events during the follow-up, even though few data are currently available [62,63].

## 6. Differential Diagnosis of Pediatric ET

A careful analysis of possible causes of reactive thrombocytosis and prolonged observation are the main steps in confronting a child with elevated platelets count. Many cases show spontaneous reduction over weeks or months. A 6-month period of observation is recommended by American and European guidelines [11,35,44,55].

Diagnostic criteria for chronic MPN have long been driven by hematological criteria and progressively adapted to the development of clinical, histological and molecular information. In 2016, they were updated into the WHO classification [4,55]. In adults, a genetically acquired driver mutation is found in the vast majority of ET cases, while this is seen only in a small percentage of children [15]. The proliferation of megakaryocytes, as well as of other myeloid progenitors, is driven by the constitutive activation of JAK/STAT2 pathways, independently from the regulated activity of growth factors (erythropoietin, thrombopoietin and its receptor c-MPL), owing to activating mutations in *JAK2*, and *MPL* genes or to abnormalities of the *CALR* gene (usually, insertions or deletions) [55]. 

In 2015, a series of 89 Italian children affected by persistent thrombocytosis were reported [15] and diagnosed with ET by WHO criteria [15]. In 2019, Ianotto et al. [43] systematically reviewed 396 pediatric cases. Kucine [44] analyzed MPN in children, adolescents and young adults. Molecular, as well as clinical features of these reports are quite similar, showing the presence of clonal markers in only 25–40% of cases. All three driver mutations were found also in pediatric cases [14,15,16,17,64,65,66]. Moreover, remarkably similar clinical features and incidences of complications were found (Table 3).

Interestingly, the relative proportion of the three driver mutations is similar to the proportion found in adults in all reports. We therefore suggest that clonal ET in children is presumably the same disease of adult age. On the other hand, the nature and the clinical significance of cases without clonal markers remains elusive. No clinical or hematological differences between clonal and non-clonal children exist.

Histological features characteristic of adult MPN have been described in children [8,67], but only half of the reported patients in all series have undergone bone marrow biopsy. This procedure, even if invasive, has demonstrated to be necessary for the correct classification of 3NEG cases. Higher megakaryocyte (MK) density, loose MK clusters and occasional grade-1 reticulin fibrosis were found. These aspects were useful to rule out reactive cases and to classify the type of MPN.

The approach to 3NEG, non-clonal histologically proven ET in a young child requires caution, mostly regarding cytoreduction and long-term follow-up. We and other Italian pediatric hematologists [11] have suggested that these cases should be addressed as “sporadic” thrombocythemia because their real malignant nature still needs to be confirmed. Other somatic mutations have been described in genes related to the epigenetic regulation of myeloid precursors proliferation.

The use of recent techniques such as next-generation sequencing (NGS) has defined panels of genetic abnormalities peculiar to MPN (comprising genes such as *TP53*, *SF3B1*, *SRSF2*, *TET2*, *PTPN11*, *IDH2*, *EZH2*, *DNMT3A* and others).

This has helped to identify prognostic differences and to build a mutational-enhanced prognostic survival risk score (MIPSS) for ET and PV in adults [68]. However, only a few reports have studied this issue in children. Pediatric MPN cases have been confirmed to harbor a poorer mutational landscape than adult ET [69,70]. We are now screening all primary ET observed at our center by the described panel [68], and no additional mutations have been found so far in 10 analyzed cases. Further analyses, such as gene expression profiling [71], might also be helpful in characterizing 3NEG cases in children.

It is possible that some pediatric 3NEG ET might be a different condition because its prevalence is too high in this age group [55,63,72]. In fact, four cases of spontaneous remission of 3NEG thrombocytosis after many years were reported [15,73] and some cases of long-lasting RT can lead to the overestimation of ET diagnosis. Therefore, these rare patients require a thorough examination comprising molecular studies, histological analysis and the application of the best available techniques to precisely define each case.

## 7. Clinical Features and Prognosis

Symptoms and complications of ET in children seem to be more benign than in adults (Table 3). Some pediatric patients are completely asymptomatic over a long time; others suffer from microvascular symptoms (headache, abdominal pain or paresthesia), while others have epistaxis or minor hemorrhages. This also occurs in children with extremely high platelet counts (up to 4500 × 10^9^/L).

In general, ET is a chronic neoplastic disease with a very long survival, exceeding 30 years for patients younger than 60 years old.

Thromboses are the main complications in adult ET patients [2,72,74], affecting morbidity and mortality such that the main goal of treatment is to prevent vascular thrombosis [55,75] rather than to correct blood counts. In ET, current risk stratification includes four categories (very low, low, intermediate and high risk) based on a validated scoring system comprising age, thrombosis history, the presence of a *JAK2*V617F mutation and the association of these factors. Cardiovascular risk factors, including smoking, hypertension or diabetes, predict arterial risk, while male gender is considered a risk factor for venous thrombosis [59]. In contrast, *CALR*-mutated ET seems to be associated with a lower thrombotic risk compared to *JAK2*V617F-mutated patients [60]. In addition, the presence of extreme thrombocytosis (>1000 × 10^9^/L) may be associated with acquired von Willebrand syndrome (VW) and, therefore, risk of bleeding [55], but not so much with thrombosis [76].

Only 4% of children with ET studied by our group [15] and of those reviewed by Ianotto et al. [43] had major thrombotic complications. Two cases had Budd–Chiari syndrome, and other similar case reports have been published [77,78]. Thrombosis of the splanchnic veins is described in young women, suggesting the possible influence of the *JAK2*V617F mutation [79]. ET children who are *JAK2*V617F-positive seem to have more frequent thrombosis than in adults, reinforcing the suggestion of clonal pediatric ET being a similar condition to adult ET [80]. Splenomegaly is also frequently observed (50–60%) in 3NEG children. However, this is not a sign of malignancy because it is a common finding in hereditary thrombocytosis with MPL mutations [19].

Leukemic transformation and evolution into myelofibrosis are rare (seven cases of MF, 1.8%, in the review by Ianotto et al. [43]) and have been mostly described in old pediatric reports when aggressive treatment was applied. However, the life expectancy of these patients should be preserved as much as possible, thus avoiding any exposure to the risk of cancer [81].

## 8. Managing Thrombocythemia in Children

Therapeutic guidelines for adults with ET are aimed to prevent vascular complications without increasing the risk of transformation [75]. Therefore, disease management is based on the risk stratification for thrombosis that has been internationally validated [60,68]. High risk factors, such as >60 age and a previous thrombotic event, are irrelevant or rare in children [80]. Thus, an identical risk-stratified strategy does not fit completely. To borrow from the experience gained with adults, ET represents a questionable option, also considering the different frequency of driver mutations and the high number of 3NEG, non-clonal cases in children in comparison to adults.

Few studies evaluating the best clinical approach to children with ET exist and, currently, only suggestions or guidelines based on experiences are published [12,18,35,44]. Moreover, any pharmacological treatment should theoretically be administered for a long time, or even for life. Long-term effects on growth, fertility and possible transformation must be weighed against all other options.

In 2015, the Italian Association for Pediatric Haematology Oncology (AIEOP) developed consensus-driven guidelines for diagnosis and treatment, available online [11]. Interestingly, even before the description of *CALR* mutations, those guidelines and criteria fit the most recent indications of Tefferi and Barbui [55]. The main criterion was to avoid any unnecessary drug administration: the initial aggressive therapeutic approach that was historically seen in many children (47% of Italian patients and most of the cases reported before 2008) has been changed. In asymptomatic children, a wait-and-watch approach is considered the best managing option. This requires careful, strict hematological and clinical observation, and so far (with a follow up of 3 to 30 years), no further complications have been described in our series. On the other hand, any thrombosis is a major event for children: would it be better to avoid any deep thrombosis rather than intervening after its occurrence? Moreover, the ability of anti-aggregation or cytoreduction to prevent major thrombosis in a child is still largely unknown.

Certainly, behavioral, familial or constitutional risk factors such as smoking, obesity, hyperlipemia and diabetes (as well as pregnancy [82]) need to be weighted in the final decision.

### 8.1. Antiaggregating Agents

Over the years, the efficacy of low-dose Acetylsalicylic acid (ASA) in PV [83] induced a wide use of similar treatments in ET, and low-dose aspirin is commonly used in adults with MPN [84]. However, a recent paper showed that the cardiovascular prophylaxis with 100 mg of aspirin appears inadequate in reducing platelet activation in most ET patients, which questions the classically used dosage [85]. Different schedules (BID/TID) are suggested for resistant symptoms [76].

Microvascular symptoms are common in pediatric ET and can be very disturbing. ASA can be used to control headache or erythromelalgia and doses can be tapered to the minimum according to efficacy. In fact, 1 mg/kg is usually effective for the control of symptoms. Caution is to be used in case of infection by varicella-zoster and influenza virus (for the possibility of hepatotoxicity and Reye syndrome). Moreover, the frequent observation of acquired von Willebrand (VW) disease, especially with extreme platelet counts (>1500 × 10^9^/L), presents hemorrhagic risk. However, it is a common observation in our pediatric series that very high platelet counts are better tolerated by children and adolescents, with a low frequency of even minor hemorrhages.

Antiaggregating agents are suggested for low-risk, *JAK2*-mutated adult ET. Since, in our experience, *JAK2* mutation is also associated with microvascular symptoms and at times major thrombosis, this recommendation can also be used for pediatric ET. It may be helpful to monitor VW ristocetin cofactor activity levels, which should be >20%, and high molecular weight multimers [86].

### 8.2. Anticoagulants

The use of anticoagulants is suggested in both adults and children with deep vein thrombosis. A recent systematic review of antithrombotic treatments adopted in patients with MPN and a history of VTE showed that direct oral anticoagulants (DOACs) and vitamin K antagonist (VKA) have a comparable, relatively low risk of recurrent thrombosis and bleeding events [87]. In addition, recent guidance suggests the use of DOACs for the treatment of splanchnic thromboses in non-cirrhotic patients in the absence of severe liver dysfunction [88]. Venous thromboembolism (VTE) is, fortunately, extremely rare in children with ET, but some cases of Budd–Chiari syndrome and cerebral sinus thromboses have been reported [77,78]. Approved anticoagulant drugs and management strategies in children with VTE have been reviewed recently [89]. The Einstein-Jr phase 3 study shows that body-weight-adjusted Rivaroxaban provides appropriate treatment for children with VTE [90].

### 8.3. Cytoreductive Drugs

The recommendations from the European Leukemia Net (ELN) reserve cytoreductive drugs for intermediate/high-risk patients with ET [75]. Hydroxyurea (HU) and interferon-alpha (IFN-a) or pegylated IFN alpha (Peg-IFNa) are considered first-line therapies for ET at any age. Initially, cytoreductive drugs such as HU, anagrelide and IFN-a were quite commonly used in thrombocythemic children even in the absence of high-risk factors. Careful analysis of the literature and of the national experiences led to a more careful, risk-adapted therapy. Therefore, high-risk children who have already experienced a thrombotic event (usually the first event leading to diagnosis) need cytoreduction, together with the addition of antithrombotic drugs [89]. Cytoreduction can be also considered in the case of low-risk ET children with resistant vascular symptoms and/or bleeding tendencies under ASA therapy [11,44,63,80]. In these cases, the reduced number of platelets to be obtained is still unknown.

Because the necessary duration of cytoreduction is unknown—yet probably very long—the histological modification of bone marrow and cytogenetical analysis should be monitored periodically in young patients undergoing cytoreduction.

There exists insufficient clinical data to recommend a specific agent in children. Moreover, no clear preference emerges among the available drugs. In the cases that need it, individually tailored treatment must be adopted. The child and the parents must be informed about the risk/benefit ratio of each choice, both interventional and observational, with a clear and thorough discussion regarding the possible long-lasting adverse effect of drugs adopted for therapy. Therefore, efforts should be made to summon prospective collaborative studies to evaluate types of drugs, duration and endpoints of treatment for ET in children.

#### 8.3.1. Hydroxyurea (HU)

HU is usually considered the first-line treatment for any MPN and is usually the benchmark to confront any other available drug [91]. HU requires chronic oral administration and is generally well-tolerated. Its major concern is the possible risk of inducing transformation into acute leukemia [92]. This, however, has not definitively been ascertained. Experience suggests that HU is efficient and safe in young adults with ET [93] and that it does not induce adverse effects on growth and fertility when used in children with sickle-cell anemia [94]. However, HU might have different (and detrimental) effects in lifelong treatment for patients with a clonal diseases such as ET.

#### 8.3.2. Interferon-Alpha (IFN-a)

IFN-a has been used since many years, but only with the introduction of its pegylated form has its side effects become more acceptable. IFN-a is capable of reducing platelet count; it is not leukemogenic nor teratogenic. PEG-IFN-a has been shown to be effective in PV resistant to HU [95,96], but results regarding ET are still controversial [97]. The possible ability of IFN-a to induce molecular and histological remission has encouraged its use [98,99].

Recently, a series of 13 children and young adults, six ET and seven PV, has been reported, where Peg-IFN-a has obtained acceptable results, with eight patients still on treatment. However, three complications were observed, and five patients discontinued treatment. Some collateral effects persist, including intolerance but also lack of complete response, and in three cases major complications were the reasons for discontinuation. Three cases showed a reduction in *JAK2*V617F allele burden [44].

#### 8.3.3. Anagrelide

Anagrelide, a nonchemotherapeutic cytoreductive drug, is an alternative to HU. It is tolerable in the long-term, but anemia occurs in about 25% of patients, and it has been suggested that it could decrease the myelofibrosis-free survival [91,100]. However, Anagrelide in adult patients with ET has been considered useful and suitable for the prevention of thrombotic complications [101]. Therefore, it has also recently been widely used in younger patients [102] and was successful mainly in children poorly compliant to HU or IFN-a.

### 8.4. JAK2-Inhibitors

Ruxolitinib is the first developed *JAK2* inhibitor, mainly used for MF and recently approved for HU-resistant PV [97,103]. In children, there exist some experiences in different conditions such as post-transplant graft versus host disease [104], and dose-finding studies have been carried out in pediatric neoplasms [105]. One infant with *JAK2*-positive polycythemia and Budd–Chiari syndrome was treated with success [106].

## 9. Conclusions

The following main conclusions are worth mentioning:

(1) The diagnosis of ET is rare but not impossible in children. It requires: (i) extensive evaluation of any possible reactive condition; (ii) studies by molecular and histological methods; and (iii) prolonged follow-up.
(2) The diagnostic WHO criteria for ET can be used in children as in adults; in addition, pediatric diagnostic guidelines should consider the possibility of familial hereditary cases.
(3) Children with confirmed ET rarely have thrombotic and hemorrhagic events and can be considered as low-risk patients.
(4) In asymptomatic children, a wait-and-watch approach is the best managing option.
(5) Cytoreductive drugs in children with ET should only be prescribed in selected cases. To date, there exists insufficient data to recommend a specific agent in children and the choice should be individually tailored.
(6) Collaborative efforts are needed to study ET and other MPN in children and adolescents, to follow and better understand their clinical and hematological behavior.
(7) Finally, therapeutic approaches, which are widely harmonized, need to be confirmed by cooperative studies.

## Figures and Tables

**Table 1:** WHO diagnostic criteria for essential thrombocytemia. Diagnosis of ET requires all 4 major criteria or the first 3 major criteria and the minor criterion to be met [3]. Thrombocytosis should be re-evaluated within at least 1 month.

**MAJOR CRITERIA**
Platelet count ≥ 450 × 10^9^/L
2. Bone marrow biopsy showing proliferation, mainly of the megakaryocyte lineage, with increased numbers of enlarged, mature megakaryocytes with hyperlobulated nuclei. No significant increase or left shift in neutrophil granulopoiesis or erythropoiesis and very rarely minor (grade 1) increase in reticulin fibers.
3. Not meeting WHO criteria for *BCR ABL1*+ CML, PV, 4. MF, myelodysplastic syndromes, or other myeloid neoplasms.
5. Presence of *JAK2*, *CALR* or *MPL* mutations.
**MINOR CRITERION**
Presence of a clonal marker or absence of evidence of reactive thrombocytosis.

**Table 2:** Causes of reactive/secondary thrombocytosis in children (adapted from [18]). The Table reports examples of significant pediatric conditions.

Infections
Inflammations Kawasaki disease Rheumatic diseases Coeliac disease Nephritis, pancreatitis
Surgery, trauma, burns, blood loss
Malignancies
Anemias Iron deficiency Hemolytic anemias
Allergic reactions
Drugs Steroids Vincristine Miconazole Antibiotics (Imipenem, Beta-lactams)

**Table 3:** Comparison of pediatric ET cases as reported by Italian Association for Pediatric Haematology Oncology Italian (AIEOP) series [15] and by a systematic review [43]. Plts = platelets number, pts = patients. (m = month, y = year).

	AIEOP Series [15]	Review [43]
# of patients F/M	89 1.8/1 (65%)	396 1.3/1 (58%)
Median age (range)	7 years (6 m–17.5 y)	9 years (6 m–20 y)
Plts × 10^9^/L	503–4400	450–4500
Microvascular symptoms	30%	23%
Splenomegaly	38%	55%
Hemorrhagies	9% minor	4.8%
Thrombosis	3.3% (clonal ET)	4% (venous > arterial)
Transformation	No	2% (MF)
Clonal cases (%)	23/89 (25.8%)	388 (40%)
*JAK2*V617F + Allele burden (%)	14 (15%) 26.2	130 (33%) 24.2
*MPL*W515L (56 pts tested)	1	4 (1%)
*CALR* mutations (74 pts tested)	6 (6.5%)	23 (6%)
X-CIP test pos (23 females tested)	6 (2 *JAK2*V617F; 2 *CALR* pts)	Not known
Marrow Biopsy (done)	45 (50%)	52%

## Data Availability

All data referred to in this review article are published and the reader is referred therein for full data availability.
